# An agent-based model of urban insurgence: Effect of gathering sites and Koopman mode analysis

**DOI:** 10.1371/journal.pone.0205259

**Published:** 2018-10-05

**Authors:** Maria Fonoberova, Igor Mezić, Jadranka Mezić, Ryan Mohr

**Affiliations:** 1 Aimdyn, Inc., Santa Barbara, CA 93101, United States of America; 2 University of California Santa Barbara, Santa Barbara, CA 93106, United States of America; Pavol Jozef Safarik University in Kosice, SLOVAKIA

## Abstract

The paper investigates the effect of preferential gathering sites on urban insurgency in an agent-based model (ABM). The ABM model was proposed in earlier work and has been validated using FBI data. There is a nonlinear tradeoff between the local density of citizens due to the number of preferential gathering sites and the ability of law enforcement officers (LEOs) to adequately patrol that leads to a non-monotonic behavior in the number of large scale outburst of insurgency with respect to the number of gathering sites. The inclusion of a moderate number of sites decreases the number of large-scale outbursts. Having no gathering sites or a large number of gathering sites has a dilutive effect on the number of large-scale outbursts. Thus, this non-monotonicity indicates that a small number of organized units produces a larger insurgency effect than a larger number of distributed units. It is also shown, using Koopman mode analysis, that the spatial morphology of agents due to the gathering sites gives rise to temporal organization of the model dynamics; there is a prominent quasi-periodic component in the number of active and intimidated citizens and in the spatial distribution of the LEOs.

## 1 Introduction

Agent-based modeling and other computer-based dynamical simulations have been increasingly used since the 1990s in the social sciences [1] as a means of understanding social processes and dynamics. These modeling efforts enable researchers to test and develop theories in a way that might not be possible using analytic and experimental methods. For example, properties of the inhabitants of a simulated world such as “fear”, “grievances”, and “ethnocentrism” can be manipulated in a simulation in a way that would not be permissible for ethical reasons in an experiment [2]. Thus, in agent-based modeling, the researcher builds an artificial environment that represents a simplified version of the real world processes of interest, and then observes the consequences of manipulating key input variables on attitudinal and behavioral outputs. Agent-based modeling has proved especially useful in understanding complex social dynamics, notably those involving interactions between micro and macro processes and the development of emergent behaviors, such as racial segregation; innovations in human organizations; civil unrest and ethnic conflict; population movement; and the diffusion of innovations and fads [2–6]. In many of these applications, the central issue addressed is the way in which agents respond to their social context, specifically to how others around them are acting, and to the efforts of organized entities to influence them through either punitive or persuasive mechanisms of social control [4]. While the theories addressed by agent-based models generally involve complex macro-level social processes, the models themselves are grounded in the actions and interactions of individual agents or groups of agents [3, 7]. Indeed, it is a guiding principle of agent-based modeling that many social behaviors emerge from these local dynamic interactions rather than being shaped from above by sociostructural forces [3, 5]. In addition, the underlying assumption of these models, at least initially, is that agents employ very simple and local behavioral rules. Thus, the goal of agent-based models is to “explore the simplest set of behavioral assumptions required to generate a macro pattern of explanatory interest” (see [5], p.146).

In previous work on agent-based models of insurgency (e.g. [2]), the role of environmental morphology was not explored. Namely, agents become active/insurgent based on association by chance and proximity in space. However, various studies have shown that environment and geography play an important role in influencing violence and terrorism [8–13]. In this paper, we use agent-based models to explore how the complex dynamics of interaction between urban insurgency forces and peace-keeping forces are modified when insurgency is organized. One of the paper’s main contributions is the introduction of Koopman Mode Analysis (KMA) [14–18] in the analysis of such models. Usually, dynamically evolving, spatially extended models are typically studied using time-simulation techniques, where no spatial or temporal reduction is deployed. KMA, on the other hand, is a mathematical method that can be applied to any stochastic, spatially extended model to identify spatial signatures (modes) with coherent temporal behavior.

A basic ingredient in the organization of an insurgency is the selection of gathering sites—namely, physical places where insurgents congregate. We add this ingredient to the model proposed in [2] in the form validated by FBI data in [19]. This addition leads to some surprising outcomes. Namely, there is a prominent, quasi-periodic component that appears upon close examination of the temporal data. What is interesting is that spatial morphology (gathering sites) leads to temporal coherency (underlying periodicity). This connectivity is unraveled by the Koopman Mode Analysis technique.

The paper is organized as follows. Section 2.1 gives an overview of the ABM model with preferential gathering sites included and defines the outputs of the simulations we are interested in. Section 2.3 gives an overview of the Koopman Mode Decomposition. In section 3.1, we investigate the effect the number of preferential gathering sites and the lattice size have on the outputs of interest. In section 3.1.1, we introduce an additional variation and study the effect the “attractiveness” of gathering sites has on the simulation outputs. This is done by varying the probability that a citizen will either move directly to a gathering site it can see or perform a random walk on its neighborhood. In section 3.2, we apply the KMD techniques introduced in section 2.3 to the ABM model in order to investigate the spatio-temporal behavior of the distributions of active citizens, intimidated citizens, and LEOs. Concluding remarks are contained in section 3.2.

## 2 Approach and methods

Agent-based models approximate real-world phenomena using agents and simple interaction rules. When one is trying to determine the causative factors of some emergent behavior, the challenge is to determine the simplest set of interaction rules that lead to the emergent phenomenon. In other cases, the interaction rules are derived from known interaction behaviors and the resulting emergent global behavior (if any) is the quantity of interest. Agent-based models complement existing methodologies and allow the exploration of situations that may be infeasible in the field.

### 2.1 Agent-based model (ABM)

The development of our model follows the work of Epstein [2] and Fonoberova et al [19]. Epstein’s work introduced an agent-based computational approach to model the situation in which a centralized authority tries to suppress a decentralized insurgency. Fonoberova et al built upon this model and validated it against crime data from 5,660 US cities. In this paper, we will build upon the model from [19].

There are two kinds of agents in the model: citizens and law enforcement officers (LEOs). Citizens are civilian members of the population who can join an insurgency. In contrast, LEOs are forces of the authority whose function in the model is to detect insurgency and attempt to curtail it. Citizens can become active in insurgency, or stay quiescent depending on a number of factors described below. LEOs always seek out active citizens. An interaction between a LEO and an insurgent may lead to cessation of the citizen’s insurgent activities. As a consequence of such “intimidation”, such a citizen stays quiescent for a randomly assigned period of time—the “intimidation term”. The intimidation term takes values between 0 and *J*_*max*_. An intimidation term of 0 would correspond a LEO issuing a warning that was immediately ignore. Longer intimidation terms encapsulate criminal justice processes such as detaining, charging, trials, conviction, etc.

**Fig 1:**
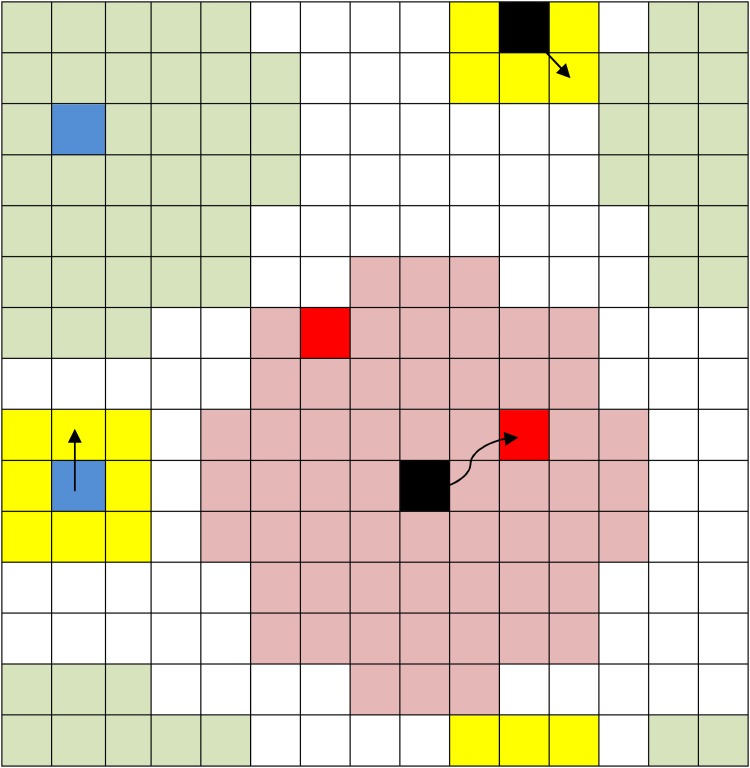
Sketch of the model. Currently quiescent (active) citizens are represented by blue (red) cells, while LEOs are represented by black cells. Yellow cells show the Moore neighborhood for an agent. Green cells show a vision of radius 4 for the citizen (blue cell). During a move, an agent inspects every cell within its vision radius (green). If there is a preferential gathering site in the vision radius, then the active agent moves in his/her Moore neighborhood in the direction of the closest site with probability P (in a simulation, P is fixed at either 0%, 25%, 50%, 75%, or 100%). Otherwise, an agent picks a random cell in his/her (Moore) neighborhood and, if the selected cell is unoccupied, he/she moves there; otherwise the agent stays put. Pink cells show a vision of radius 4 for the LEO (black cell). The LEO can intimidate the nearest active citizen in its field of vision (in this model, with probability 1). If there is no active citizen, then the LEO moves to a random cell in its Moore neighborhood in the same manner that a citizen does. The citizens calculate their state depending on conditions on the lattice within their field of vision.

For simplicity, all events transpire on a lattice with periodic boundaries. All citizens move once per day, and LEOs move several times per day. We introduce preferential gathering sites on the lattice so that active citizens move in their Moore neighborhood in the direction of the closest preferential gathering site. This change introduces the effect of aggregation due to organization into the model which is important in capturing the possibility of strong, but localized, outbursts in certain areas. Fig 1 gives a schematic representation of the lattice and a brief description of how simulations are updated. For complete details of the model, consult S1 Appendix and [19].

The model as presented is a simplified representation of a complex phenomena having many dimensions. There are many other behaviors that could be added to our model and others that are implicitly captured by certain parameters. One interesting dimension that could be explicitly included is the role of groups and third-parties in peace-keeping and conflict resolution [20]. Philpot [21] built agent-based models that explored these effects. However, LEOs were not explicitly part of that model. While interesting, we chose to focus here on the interaction between LEOs and citizens. Some third-party effects, such as citizens reporting and drawing LEOs’ attention criminal activity, are implicitly captured in our model through the LEOs’ larger (relative to a citizen’s) vision radius. Here, the term *visual* should not be thought of as only physical sight, but the LEOs’ ability to collect information from a large geographic area, whether that be from citizen reports, CCTV, or other methods. We have also chosen a simple random walk model for the movement of citizens and LEOs. More natural movement patterns could be encoded in the model as is done in [22]. However, this requires the addition of visual obstacles in the environment. As we are interested in the effect of the number of gathering sites on insurgency, an inclusion of visual obstacles would present a confounding variable. Visual obstacles are a subset of a wider effect of geography on insurgency [8–12]. While an interesting future direction, agent-based models including these effects would need to be validated with each new mechanism added, and thus, we stick to the simpler validated model. Our focus is on how the number of gathering sites affects levels of insurgency and the introduction and role of Koopman mode analysis in determining these effects.

### 2.2 Model output variables

We now describe the output variables of the model; their explicit functional definition is given in Table 1. The total number of citizens active at the end of the day is denoted by *Act*; *AJ* denotes the total number of citizens active at the end of the day or intimidated during the day; *pop* is the population size; and *tp* is the length of a specific time period.

**Table 1:** Outputs of interest.

Nr.	Output Name	Output Definition
1	Number of active citizens per day in percent	$NA_{tp}=\frac{\frac{1}{tp}\sum_{tp}Act}{pop}*100$
2	Number of large-scale outbursts (revolutions) per 1,000 days	*NR*
3	Peak number of active citizens	$PNA=\frac{AMNA}{pop}*100$
4	Number of violent outbursts per year	$NV_{Y}=\frac{365}{AWT}$
5	Rate of insurgency	$RV_{tp}=\frac{\frac{1}{tp}\sum_{tp}AJ}{pop}*1000$

The percent of the population, averaged over the time period *tp*, that is active is denoted by *NA*_*tp*_. Following [2], we introduce a threshold (e.g. 5% of population) and say there is a large-scale outburst of insurgency if *NA*_*tp*_ exceeds the threshold. *NR* is the total number of large-scale outbursts (or revolutions) of insurgency per 1,000 days.

The rate of insurgency, denoted by *RV*_*tp*_, is the number of citizens active at the end of the day or intimidated during the day per 1,000 citizens for a specific time period. While this number includes LEO activity, *NA*_*tp*_ does not. Thus, the difference in trends between these two numbers reflects LEO activity; e.g. if *RV*_*tp*_ = *NA*_tp_*10, then there was no intimidation and thus no police work (compare the equations in Table 1).

Let *AMNA* be the maximum number of active citizens during the large-scale outburst of insurgency averaged over all large-scale outbursts. We denote the peak number of active citizens in percentage by *PNA*. This number indicates how large the large-scale outburst of insurgency gets.

The variable *AWT* denotes the waiting time between two large-scale outbursts of insurgency averaged over all outbursts. The number of violent outbursts per year, denoted by *NV*_*Y*_, is inversely proportional to *AWT*. Thus, a larger value for *NV*_*Y*_ indicates more frequent large-scale outbursts.

For the simulations, we considered six lattices sizes (100x100, 200x200, 300x300, 400x400, 500x500, 600x600) with the corresponding real population of 63,000; 252,000; 567,000; 1,008,000; 1,575,000; and 2,268,000. In this, we assume one “citizen-agent” represents 9 real citizens. These real population sizes give the same density of citizen-agents for each lattice. For each simulation, citizen-agents (hereafter, citizens) and LEOs were initially placed randomly on the lattice and preferential gathering sites were uniformly distributed on the lattice. Each experimental run consisted 10,101 days computed on the lattice. Each experiment was repeated at least 16 times. Each output was computed for each experimental run, and then the results were averaged over all runs.

### 2.3 Koopman Mode Decomposition

Koopman Mode Decomposition (KMD) is a way to break down the evolution of observables (quantities of interest) of an evolving system into components having simple time-dependence. Similar to Proper Orthogonal Decomposition (POD), KMD takes a time series of observables and decomposes it into a set of spatial modes having time dependent coefficients. One of the main differences is that, whereas the time-dependent coefficient of each POD mode usually contains many different frequencies, the time-dependent coefficient of each KMD mode only contains a single frequency. The spatial modes coming from KMD have simple temporal behavior—they are either exponentially damped, undamped, or growing oscillations at a fixed frequency depending on whether the corresponding frequency *w*_*j*_ is real, imaginary, or complex, respectively. Also, as opposed to POD, KMD takes into account dynamical information. POD algorithm treats a times series as a collection of points devoid of any time-dependence, whereas KMD explicitly uses the time index of vectors in the time series. As an analogy, one can think of the classical problem of decomposing a vibrating string into sinusoidal shapes (the spatial modes) each having their own vibration frequency (the temporal component). KMD can be thought of as an extension of this idea to much more general and abstract systems.

Let L={(i,j)|1≤i,j≤LD} be the simulation lattice, where the lattice dimension *LD* is either 100, 200, 300, 400, 500, or 600. The evolved state of the simulation at time *t* is the 3-dimensional vector denoted by *X*(*t*, *i*, *j*, *ξ*) = (*x*_1_(*t*, *i*, *j*, *ξ*), *x*_2_(*t*, *i*, *j*, *ξ*), *x*_3_(*t*, *i*, *j*, *ξ*)). Here *ξ* denotes a sequence of random numbers that are used to select particular LEOs or citizens at each simulation step; whether a citizen moves to a preferred gathering site or performs a random walk on its Moore neighborhood; and determine the next location of a citizen’s random walk; etc. The first component *x*_1_(*t*, *i*, *j*, *ξ*) of the evolved state vector determines the type of agent at location (*i*, *j*) in the lattice at time *t*; *x*_1_(*t*, *i*, *j*, *ξ*) = 0 denotes there is no agent at location (*i*, *j*), *x*_1_(*t*, *i*, *j*, *ξ*) = 1 denotes a citizen is present, and *x*_1_(*t*, *i*, *j*, *ξ*) = 3 denotes there is a LEO there. The second component *x*_2_(*t*, *i*, *j*, *ξ*) denotes the state of the citizen present at (*i*, *j*) at time *t*; *x*_2_(*t*, *i*, *j*, *ξ*) = 0 if there is a LEO or no citizen present, *x*_2_(*t*, *i*, *j*, *ξ*) = 1 if citizen is quiescent, *x*_2_(*t*, *i*, *j*, *ξ*) = 2 if citizen is active, add *x*_2_(*t*, *i*, *j*, *ξ*) = 3 if the citizen is intimidated. Finally, *x*_3_(*t*, *i*, *j*, *ξ*) denotes the intimidation time of the agent; it is 0 if there is no citizen, there is a citizen that is not intimidated, or there is a LEO present. The state space is $X={\left(R^{3}\right)}^{LD\times LD}\times \Omega$, where *LD* is the lattice dimension. ${\left(R^{3}\right)}^{LD\times LD}$ is a tensor holding the states of all the agents, and Ω denotes the space of all sequences, *ξ*, of *p*-dimensional random vectors, where *p* is the number of random numbers needed every iteration to update the ABM’s state. The components of the random vectors are independent random variables. The state *X*(*t*, *i*, *j*, *ξ*) is driven by the ABM model (see S1 Appendix); it is a map from X×Ω into X. The evolution in Ω is given by a shift on the sequences.

As all of the outputs of interest are derived from the number of active citizens per day, we describe the Koopman mode decomposition in terms of this time series. Let *NA*(*t*, *ξ*_*ℓ*_) be the number of active citizens at the end of the day at time *t* for the simulation corresponding to the ℓ^*th*^-generated sequence of random vectors $\xi_{\ell }=\left(\xi_{\ell }^{\left(1\right)},\xi_{\ell }^{\left(2\right)},\ldots \right)$. *NA*(*t*, *ξ*_*ℓ*_) is an observable on X×Ω since it computes the total number of active citizens at each instance of time; i.e., *NA*(*t*, *ξ*_*ℓ*_) = ∑_*i*, *j*_ *f*(*X*(*t*, *i*, *j*, *ξ*_*ℓ*_)) where *f* is the operation that takes *X*(*t*, *i*, *j*, *ξ*_*ℓ*_) and determines the number of active citizens at (*i*, *j*) in the lattice. Associated with the evolution of the time series *NA*(*t*, *ξ*_*ℓ*_), there is a family linear operators, called the Koopman operator family *U*^*t*^, that evolves *NA*(*t*, *ξ*_*ℓ*_) in time; i.e. $U^{s}\left[NA\left(t,\xi_{\ell }^{\left(t\right)}\right)\right]=NA\left(t+s,\xi_{\ell }^{\left(t+s\right)}\right)=\sum_{i,j}\;f\left(X\left(t+s,i,j,\xi_{\ell }^{\left(t+s\right)}\right)\right)$ (see [15] or [23] for details on the theory). Since *U*^*t*^ is a family of linear operators, we can compute its eigenfunctions and eigenvalues. As we are interested in the spatio-temporal evolution of simulation outputs, we consider a vector-valued observable $F:X\times \Omega \to R^{N}$. For example, each of the *N* components of *F*, where the *n*-th component is denoted by *F*_*n*_, determine the number of active citizens at time *t* in some specified region *R*_*n*_. In this case, *F*_*n*_ would be defined as $F_{n}=\sum_{\left\{\left(i,j\right)\in R_{n}\right\}}\;f\left(X\left(t,i,j,\xi_{\ell }^{\left(t\right)}\right)\right)$.

The evolution *F*(*t*, *x*, *ξ*) can be expanded into parts corresponding to the point spectra (eigenvalues) and the continuous spectrum of *U*^*t*^:

$$\begin{array}{c} U^{t}F\left(s,X,\xi \right)=F\left(t+s,X,\xi \right)=\sum_{\omega_{j}}e^{i\omega_{j}\left(t+s\right)}M_{j}\phi_{j}\left(X,\xi \right)+\int e^{i\theta (t+s)}dE_{\theta }F\left(0,X,\xi \right), \end{array}$$ (1)

where *X* is the vector of initial (ABM) states of the citizens and LEOs; $\phi_{j}:X\times \Omega \to C$ is an eigenfunction of *U*^*t*^, where $e^{i\omega_{j}t}$ is its associated eigenvalue with frequency $\omega_{j}\in R$ (i.e. $U^{t}\phi_{j}(X,\xi )=e^{i\omega_{j}t}\phi_{j}(X,\xi )$); $M_{j}\in C^{N}$ is the Koopman mode corresponding to *ϕ*_*j*_, θ∈R; and *E*_*θ*_ is a projection-valued measure defined on the real line. Note that any noise or randomness of the time series is contained in *E*_*θ*_. The portion of the expansion in terms of eigenvalues is termed the Koopman mode decomposition of *X*. We are most interested in the Koopman modes *M*_*j*_, which are (vector-valued) coefficients of the eigenfunctions *ϕ*_*j*_. If $F:X\times \Omega \to R^{N}$ was the vector-valued observable whose components gave the number of active citizens in different regions of the lattice, then the components of *M*_*j*_ also give a number of active citizens in each of those regions. However, *M*_*j*_ has a time dependence given by the eigenvalue $e^{i\omega_{j}t}$. The temporal behavior of any region *R*_*n*_ would be given by the time-dependent sum of the *n*^*th*^ component of the Koopman modes *M*_*j*_; that is

$$\begin{array}{c} F_{n}\left(t,X,\xi \right)=\sum_{\omega_{j}}e^{i\omega_{j}t}M_{j,n}\phi_{j}\left(X,\xi \right), \end{array}$$ (2)

where *F*_*n*_(*t*, *x*, *ξ*) and *M*_*j*,*n*_ are the *n*^*th*^ components of the *N* length vectors *F*(*t*, *X*, *ξ*) and *M*_*j*_. If there are only a few dominant modes *M*_*j*_, then the above sum can be approximated by just summing over those dominant modes. This would give a simple reduced order model of the dynamics of *F*.

To compute the Koopman mode decomposition, this paper uses the Arnoldi-like algorithm from [16].

## 3 Results

### 3.1 Phenomenology: Test case with high rate of insurgency

In previous work, we considered the question of the influence of the number of LEOs on large-scale violent outbursts [19] and observed a phase-transition type phenomenon where large-scale outbursts occurred upon decreasing the number of LEOs. Here, we consider the case when the number of LEOs per 1,000 citizens, *NL*, is selected in such a way so that large-scale outbursts happen. For all model parameters we assume the fixed values presented in S2 Appendix (for sensitivity analysis of this class of models see [24]).

**Fig 2:**
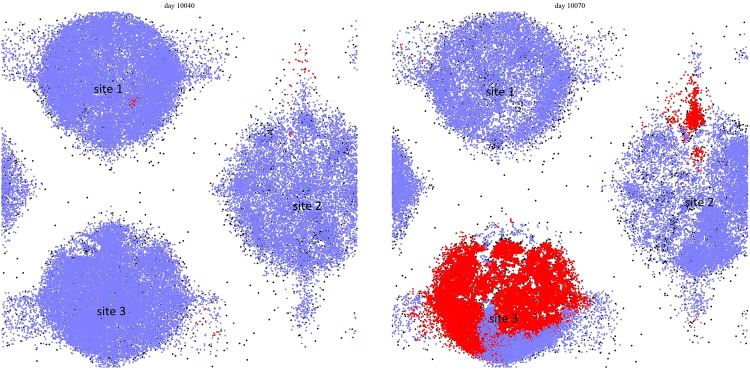
Lattice situation at day 10040 and day 10070 for the case of 3 preferential gathering sites. Citizens, who can become active, are colored blue if quiescent and red if active. LEOs are colored black, never active citizens and unoccupied sites are white.

**Fig 3:**
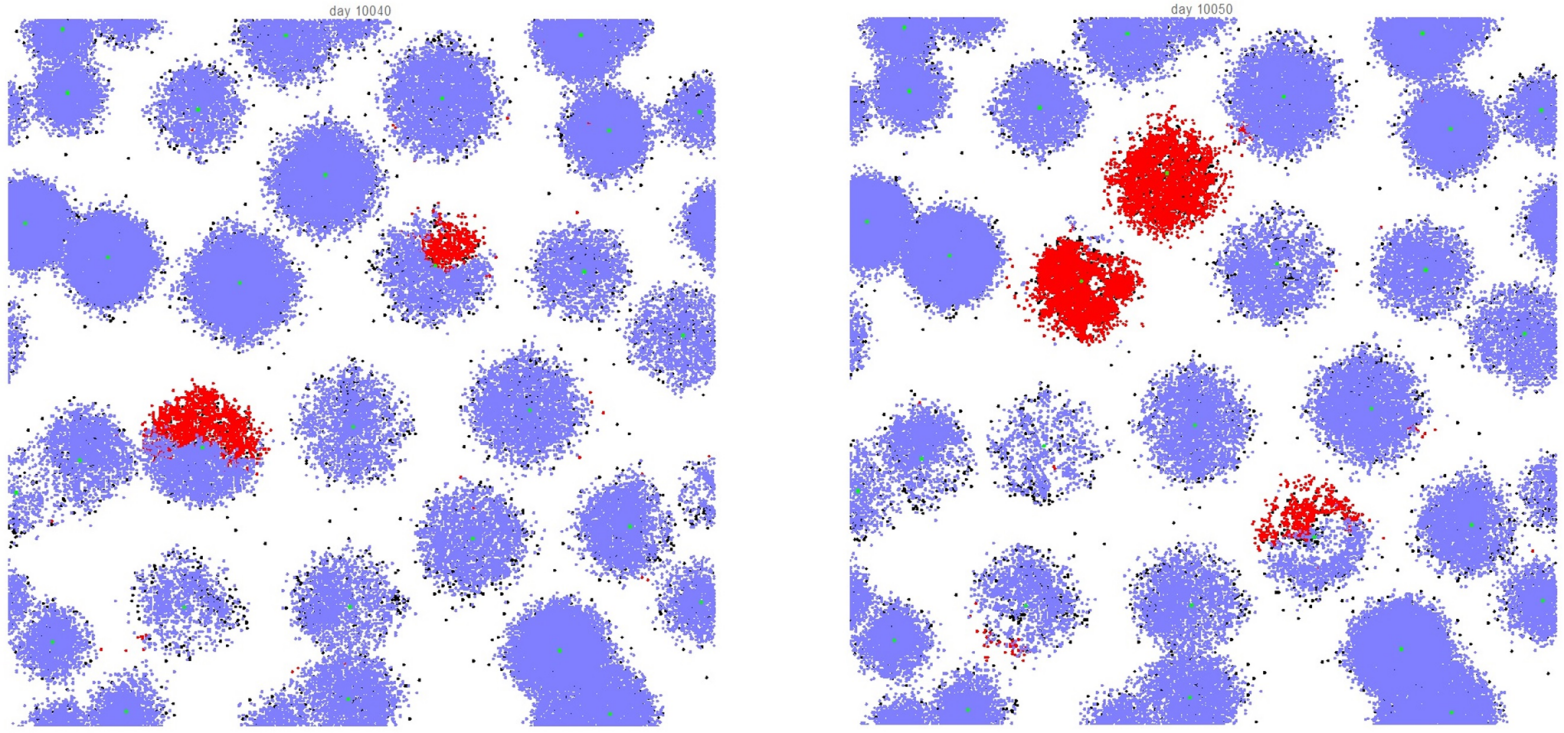
Lattice situation at day 10040 and day 10060 for the case of 30 preferential gathering sites. Citizens, who can become active, are colored blue if quiescent and red if active. LEOs are colored black, never active citizens and unoccupied sites are white.

**Fig 4:**
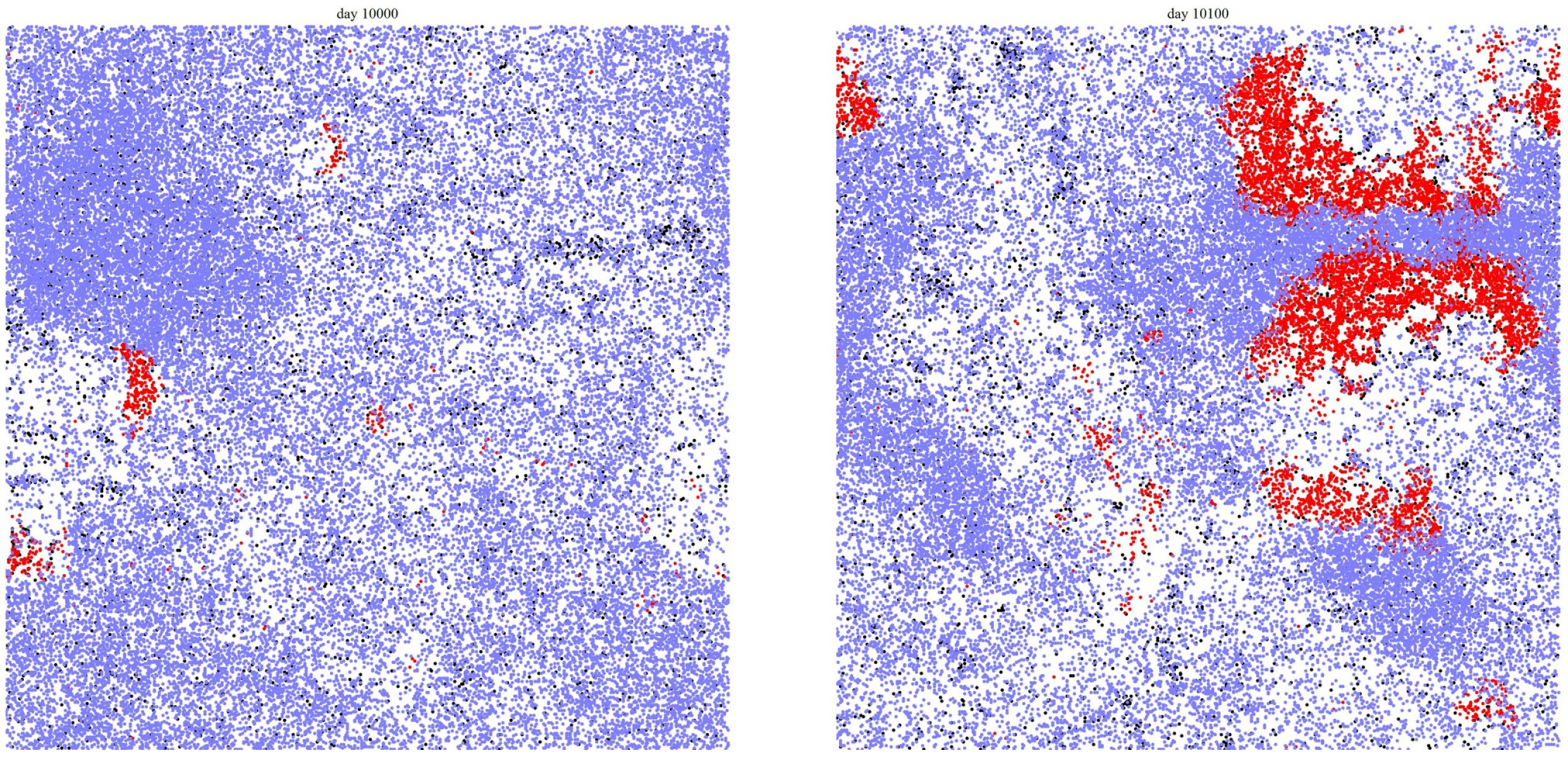
Distribution of active citizens during a violent outburst when no gathering sites are present. **(a) 10,000 days. (b) 10,100 days**. This figure is taken from previous related work [19].

The typical effect of gathering sites can be seen in Figs 2 and 3, where the spatial distributions of active (red), potentially active (blue), and inactive (white) citizens are shown for the case of 3 and 30 gathering sites, respectively. One can clearly see the effect in the non-uniform distribution that reflects the topology of the gathering site distribution. This is to be contrasted with the case in Fig 4 where we show the distribution during a violent outburst when no gathering sites are present.

**Fig 5:**
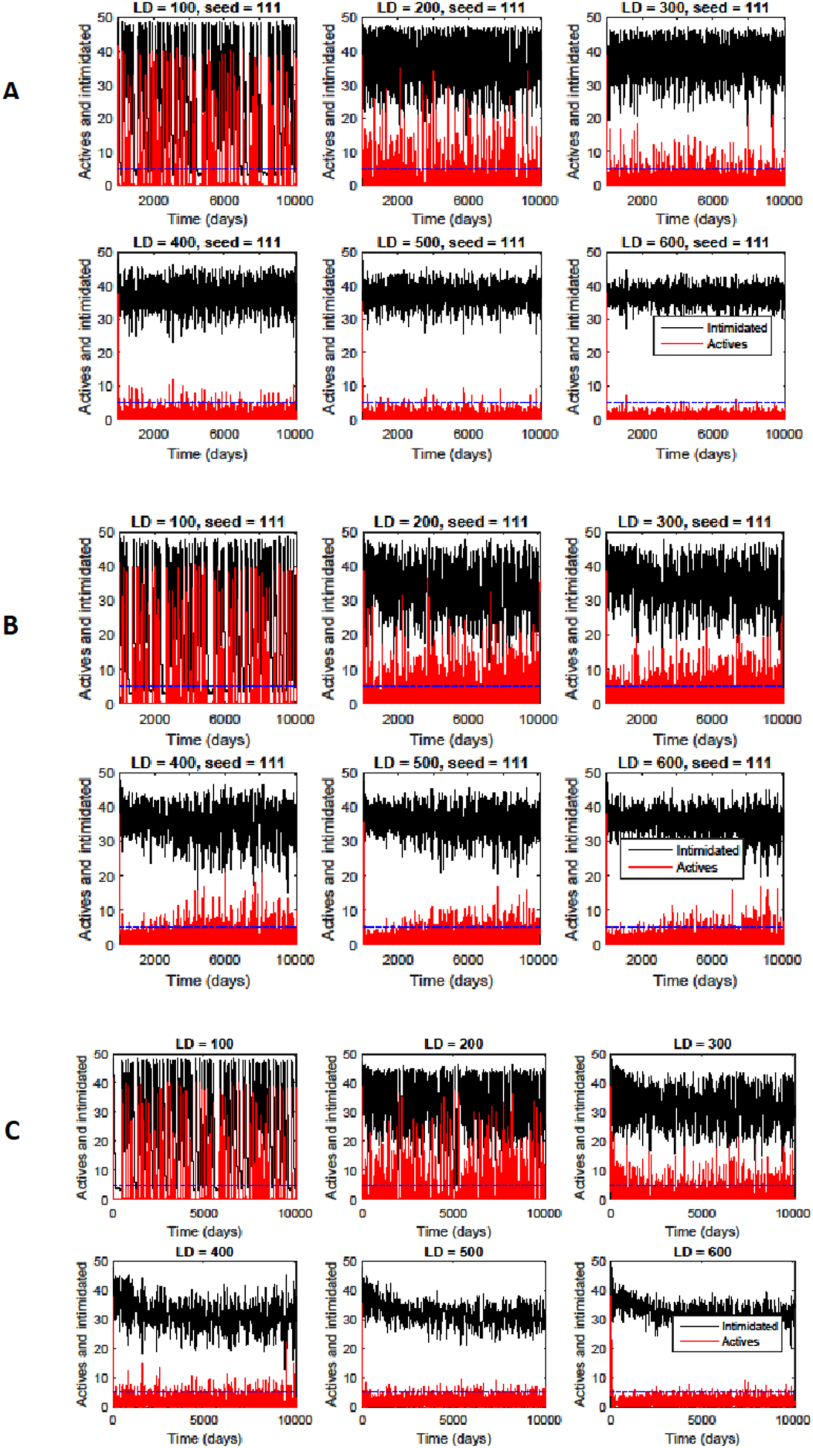
Active (red) and intimidated (black) citizens (in percent) at different lattice sizes (denote by *LD*) for the case of (A) 0 preferential gathering sites, (B) 3 preferential gathering sites, (C) 30 preferential gathering sites.

The time-evolution of the number of active and intimidated citizens is shown in Fig 5A–5C, corresponding to 0, 3, and 30 preferential gathering sites. As we can see from Fig 5A), in the case of no gathering sites, the large-scale outbursts of activity are relatively rare for big lattices (*LD* ≥ 500, where *LD* denotes the lattice size). For the smaller lattice sizes (*LD* ≤ 200), large-scale violent outbursts are common even with increasing preferential gathering sites; for *LD* = 100 and 200 there is not much difference in the level and frequency of large-scale violent outbursts. Such small lattices artificially concentrate the number of active citizens; each citizen can “see” a large portion of their environment and thus have a higher probability of getting activated by other citizens. For larger lattice sizes, each citizen only sees a smaller neighborhood of themselves and thus can only see a smaller portion of the population at any one time. The number of gathering sites has little effect on the level of large-scale violent outbursts because they are separated enough geometrically to effectively dilute the aggrieved population. Fig 5B) shows that, with the introduction of a small number of gathering sites (modeling organized activity), the large-scale outbursts of activity happen with higher probability when considering the larger lattice sizes (*LD* ≥ 400). Interestingly, when a larger number of sites of organized activity is introduced, Fig 5C) indicates that the large-scale outbursts of activity happen rarely for the larger lattice sizes. This can be explained by the fact that each organizational center (gathering sites) is attracting a smaller number of insurgents. Additionally, the gathering sites themselves are geographically separated enough so that they do not influence each other. The result is that the insurgency does not typically coalesce into a stronger outburst (see Fig 3).

**Fig 6:**
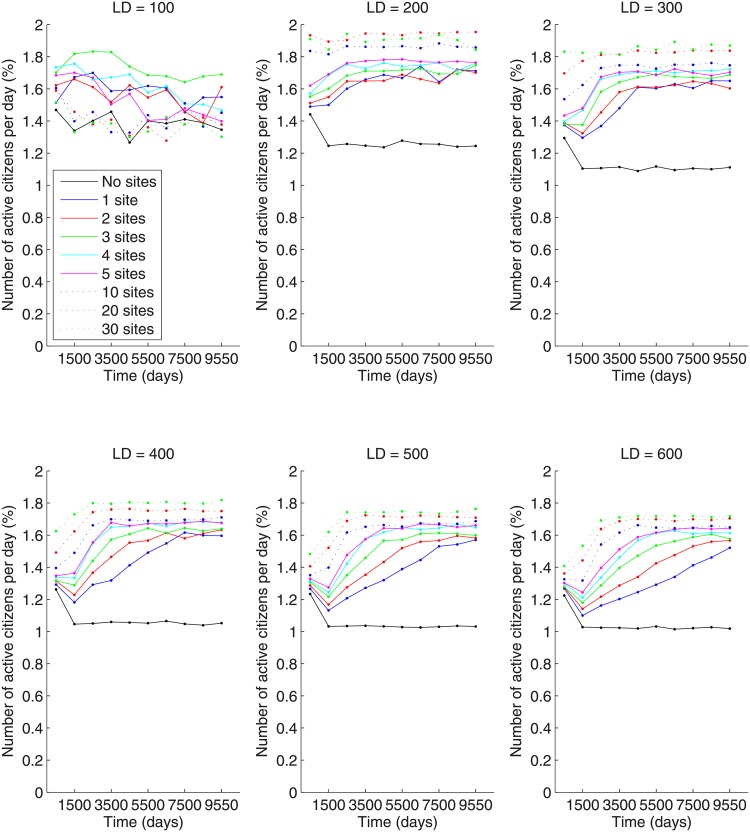
Number of active citizens per day (in percent) for different numbers of preferential gathering sites and lattice sizes of (A) 100x100 (LD = 100), (B) 200x200 (LD = 200), (C) 300x300 (LD = 300), (D) 400x400 (LD = 400), (E) 500x500 (LD = 500), and (F) 600x600 (LD = 600).

Fig 6 demonstrates the effect on the number of active citizens per day (averaged over all simulations) due to lattice size and number of preferential gathering sites. For the 100x100 lattice (LD = 100), no strong relationship in the number of active citizens can be seen between the larger numbers of preferential gathering sites (10, 20, 30 sites). As time evolves, the levels of active citizens interchange their ordering. However, the number of active citizens for these larger number of gathering sites are comparable to the case of no preferential gathering sites. For LD = 100, the results suggest a non-monotonic behavior in the number of actives; it starts low for zero preferential gathering sites, increases for modest numbers of gathering sites, and then decreases to original levels as the number of sites increases.

As the lattice size changes, a trend in the number of actives per day becomes more clear; the number of actives per day increases with the number of preferential gathering sites. Another thing to note is that for all lattice sizes and numbers of preferential gathering sites, the number of active citizens per day increases to a saturation level. Furthermore, if we look at the saturation value of the number of active citizens for the case of 30 preferential gathering sites for all lattice sizes, we see that there is a slight decrease in the number of active citizens as the lattice increases from LD 200 (200x200 lattice) to LD 600 (600x600 lattice). This appears to be the case with other numbers of preferential gathering sites, although it is not as clearly shown as in the 30 sites case. The saturation value represents the statistical equilibrium between the number of active citizens and the number of citizens intimidated by LEOs.

**Fig 7:**
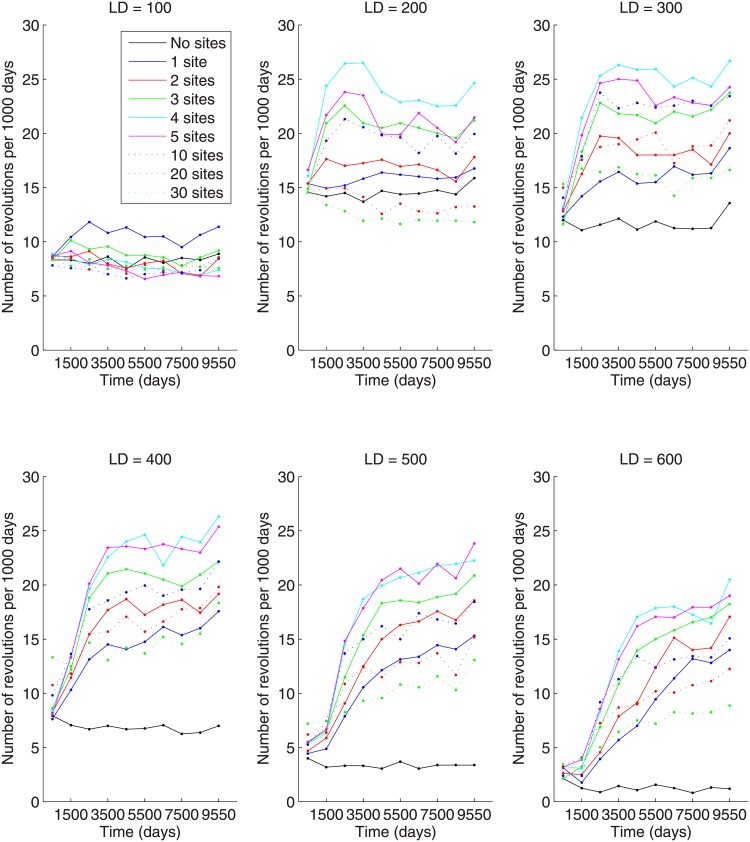
Number of large-scale outbursts per 1,000 days for different numbers of preferential gathering sites and lattice sizes of (A) 100x100, (B) 200x200, (C) 300x300, (D) 400x400, (E) 500x500, and (F) 600x600.

Another phenomenon that is clear in Fig 6, especially for the larger lattice sizes, is the monotonicity of the saturation level with respect to the number of preferential gathering sites. The larger the number of gathering sites, the higher the saturation level of active citizens. This contrasts with the non-monotonic behavior in the number of large-scale outburst with respect to the number of gathering sites. As we can see from Fig 7, the number of large-scale outbursts per 1,000 days has a peak at about 5 gathering sites. Mechanistically, as a larger number of gathering sites is introduced, the number of large-scale outbursts of insurgency decreases because the citizens are more spread on the lattice and the possibility of forming big clusters of activity is smaller. On the other hand, with a very small number of gathering sites, say between 1 and 3, there is a large concentration of citizens around the gathering site, yet there is still a small number of large-scale outbursts. In this situation, LEOs can concentrate man-power on these sites and intimidate large numbers of active citizens in order to prevent a large number of large-scale outbursts. This is also born out in Fig 5. Indeed, if we compare the number of intimidated citizens for 3 and 30 gathering sites for a fixed lattice size, we see that there is much more variability in the number of intimidated citizens. A side effect of this is that it is possible for a large number of intimidated citizens to be released back into the population at the same time and immediately become active, resulting in a large-scale outburst. We will see this confirmed below when we look at the peak number of active citizens in a large-scale outburst as a function of the number of preferential gathering sites. The decreased number of large-scale outbursts for larger numbers of preferential gathering sites is reminiscent of the common “divide and conquer” strategy for quenching insurgency [25]. The non-monotonicity is the result of an interesting trade-off in the local concentration of citizens due to the number of gathering sites (concentrations drop with increasing gathering sites) and the decreasing ability of LEOs to adequately cover an increasing number of gathering sites (less LEOs per site as the number of sites increase). Thus, this non-monotonicity indicates that a small number of organized units produces a larger insurgency effect than a larger number of distributed units.

At first pass, it may seem that the phenomena indicated in Figs 6 and 7 are in disagreement. After all, the definition of a large-scale outburst is that the number of active citizens exceeds 5% of the population. Fig 6 seems to indicate that the number of active citizens never exceeds 2%, so how can there be any large-scale outbursts? The answer lies within how each of these quantities are computed. Each simulation was run 16 times. For each run, there is a corresponding time series for the number of active citizens. For any single simulation run, there will be outbursts of activity that exceeds 5% of the population, resulting in an increase in the number of large-scale outbursts. However, these outbursts are somewhat rare. If we look at the the same time period for this large-scale outburst in other simulation runs, one will likely not see a large-scale outburst and the number of active citizens will be less than 2% of the population. When we average the number of active citizens at each time point over the number of simulation runs, the large-scale outburst is washed out by the other 15 simulations which had small percentages of active citizens during the same time period. Recall that each of the above quantities in Table 1 is computed for each simulation run first and then those numbers are averaged over the number of simulation runs.

**Fig 8:**
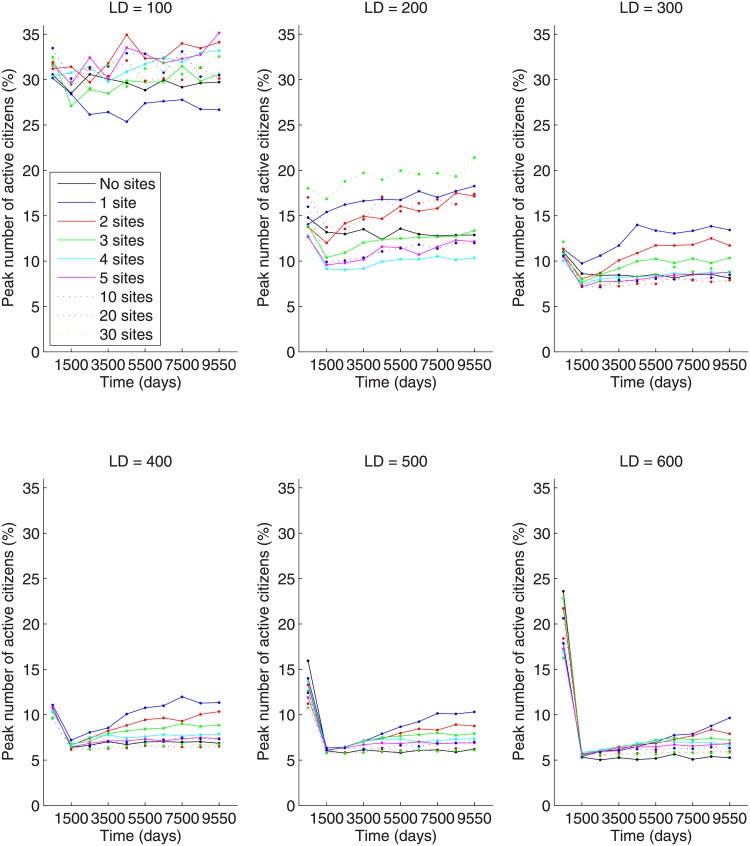
Peak number of active citizens (in percent) for different numbers of preferential gathering sites and lattice sizes of (A) 100x100, (B) 200x200, (C) 300x300, (D) 400x400, (E) 500x500, and (F) 600x600.

While the number of large-scale outbursts is non-monotonic in the number of gathering sites, the peak number of active citizens during a large-scale outburst is monotonically decreasing with respect to the number of gathering sites when the lattice size is 300x300 or greater (see Fig 8). For lattice sizes 300x300 and above, the peak number of active citizens during a large-scale outburst decreases with the number of preferential gathering sites (the case of 0 gathering sites can be regarded as a limiting case of an infinite number of gathering sites where at each step the citizen randomly chooses to go to a new site within their vision radius). As discussed above, the number of large-scale outbursts is the highest for a moderate (4 to 5) number of gathering sites, with large-scale outbursts happening less frequently for a small number of gathering sites. This is due to LEOs being able to focus man-power on one or two locations. However, when a large-scale outburst does occur in the one (or two) gathering site case, it is more severe due to the very high local density of citizens surrounding each gathering site.

Additionally, for any fixed number of gathering sites, the peak number of active citizens is largest for small lattice sizes and decreases monotonically in the lattice size. This is due to the relative vision radius of each citizen affecting their ability to find the preferential gathering sites. As the lattice size increases, the amount of area a citizen’s vision can cover relative to the lattice size decreases. This means there is a higher likelihood of a citizen not having a gathering site within their vision radius, so they continue to perform a random walk on the lattice rather than going to a gathering site. This depresses the local concentration of citizens at each gathering site, thus depressing the severity of any large-scale outburst.

**Fig 9:**
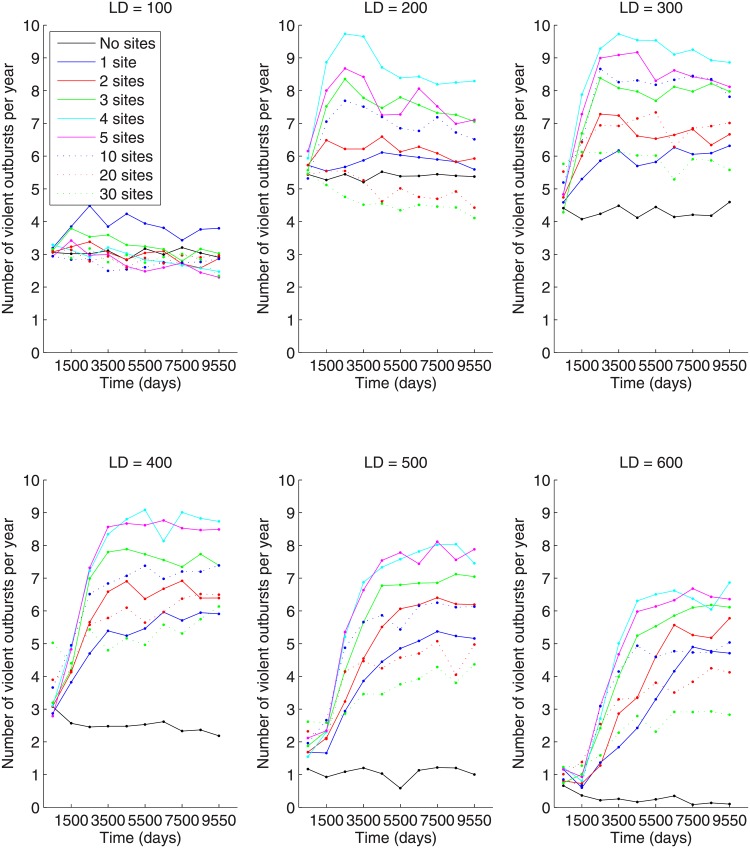
Number of violent outbursts per year for different numbers of preferential gathering sites and lattice sizes of 100x100, 200x200, 300x300, 400x400, 500x500, 600x600.

We see that the number of violent outbursts per year follows the same non-monotonicity pattern—peaking around 4 to 5 preferential gathering sites (Fig 9)—that the number of large-scale outbursts do. This is expected since the number of large-scale violent outbursts per year is directly proportional to the number of large-scale outbursts per 1000 days (see Table 1). The larger the number of large-scale outbursts per 1000 days, the shorter the average waiting time between such outbursts. The average number outbursts per year is, by definition, inversely proportional to the average waiting time between outbursts. Thus, for large numbers of introduced preferential gathering sites, the number of violent outbursts per year decreases due to the increase in waiting time between large-scale outbursts.

**Fig 10:**
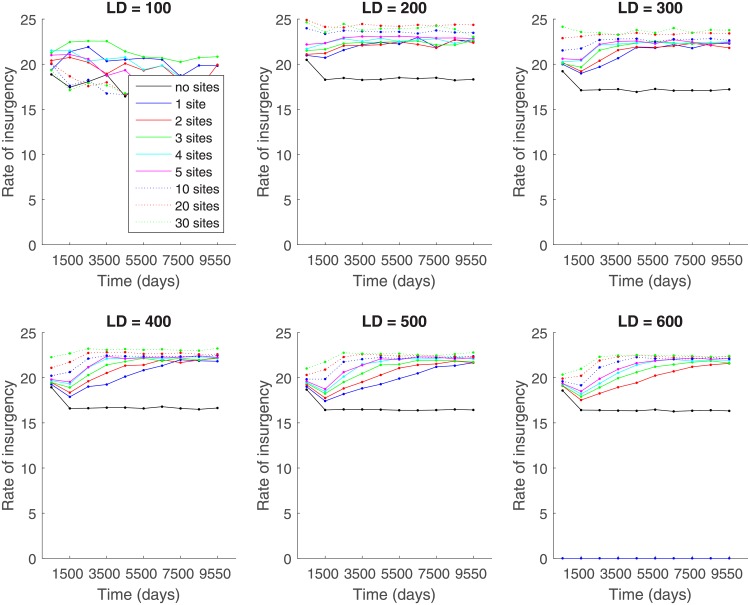
Rate of insurgency for different numbers of preferential gathering sites and lattice sizes of (A) 100x100, (B) 200x200, (C) 300x300, (D) 400x400, (E) 500x500, and (F) 600x600.

Fig 10 shows the rate of insurgency for different numbers of gathering sites and lattice sizes. As would be expected, it follows a similar trend as the number of active citizens per day does (Fig 6) since the rate of insurgency depends on both the number of active citizens at the end of the day and the number of intimidated citizens during the day. It is interesting that the rate of insurgency, when reported in the number of citizens, does not change significantly with increasing lattice sizes, but it is a decreasing quantity if reported as a percentage of the population since the real population size increases with lattice size to maintain a 70% population density.

**Fig 11:**
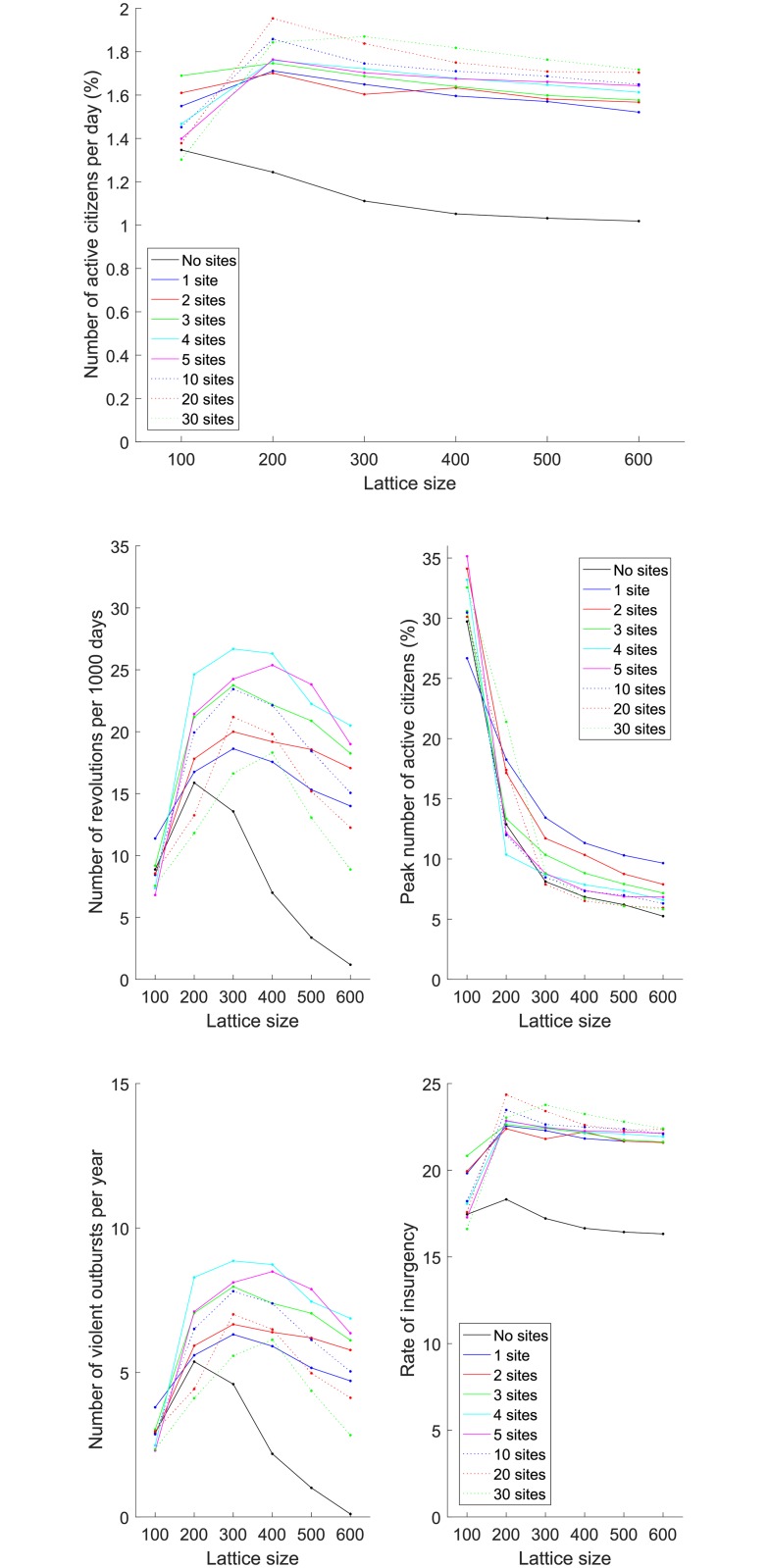
(A) Number of active citizens per day (in percent), (B) number of revolution per 1,000 days, (C) peak number of active citizens (in percent), (D) number of violent outbursts per year, (E) and rate of insurgency for different numbers of sites in dependence on lattice size. Outcomes averaged over time interval [9000;10101].

It is interesting to consider effect of lattice size on outcomes. In Fig 11, we plot the dependence of all model outcomes on the lattice size averaged over the time interval [9000, 10101], thus capturing the time period over which the dynamics have become stationary (in the statistical sense). The percentage of active citizens per day does not change essentially with the increase in lattice size. However, the number of revolutions per 1,000 days peaks for lattices of average size. This can be explained by the fact that for smaller lattice sizes large-scale outbursts are not as frequent. On the other hand, for bigger lattices, there is a lot of activity on the lattice, but because the active citizens are spread out, there are no large-scale outbursts of activity. As discussed above, the peak number of active citizens is the highest for small lattices and decreases with increasing lattice size. The number of violent outbursts per year is non-monotonic and biggest for lattices of medium size. The rate of insurgency does not differ significantly for all lattice sizes. The behavior of a number of outputs is non-monotonic in the number of preferential gathering sites leading to the conclusion that introducing a large number of gathering sites has a dilutive effect on large-scale outbursts.

#### 3.1.1 Dependence on probability of gathering

**Fig 12:**
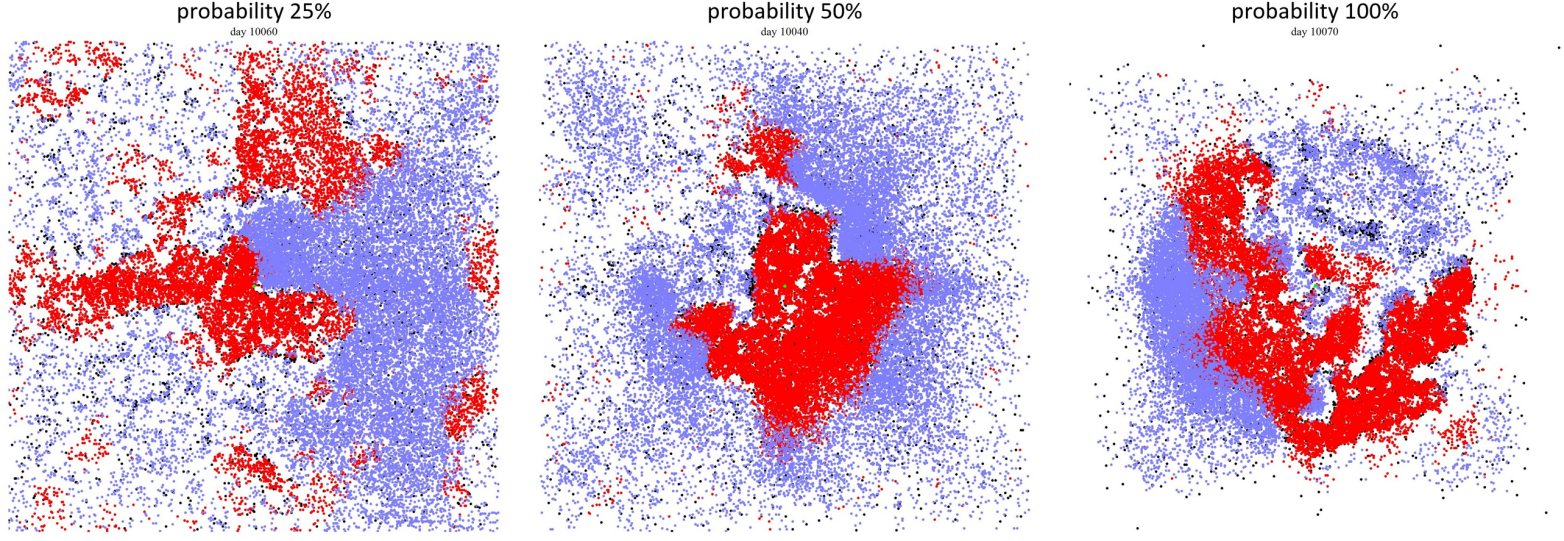
Lattice situation during the outburst of activity with (A) 25%, (B) 50%, and (C) 100% probability with which active citizens try to move to the (single) preferential gathering site. Citizens, who can become active, are colored blue if quiescent and red if active. LEOs are colored black, never active citizens and unoccupied sites are white.

In the results presented in the previous section, active citizens moved to the preferential gathering site with probability 1. Here, we model “attractiveness” of the gathering sites by introducing probabilities that determine whether a citizen will move to a gathering site in their vision radius or execute a random walk on their neighborhood. The probabilities range between 0 and 1 (probabilities are expressed in percentages in the figures) and are fixed during each simulation and uniform across citizens. Parameter values are the same as in the previous section and are listed in S2 Appendix. From the simulation outputs in Fig 12—which correspond to the case of a single gathering site—we can see, as expected, the bigger the probability is, the denser the cluster of active citizens around a gathering site becomes. We plot all model outcomes as 2D plots with respect to lattice size and probability during time [9000, 10101].

**Fig 13:**
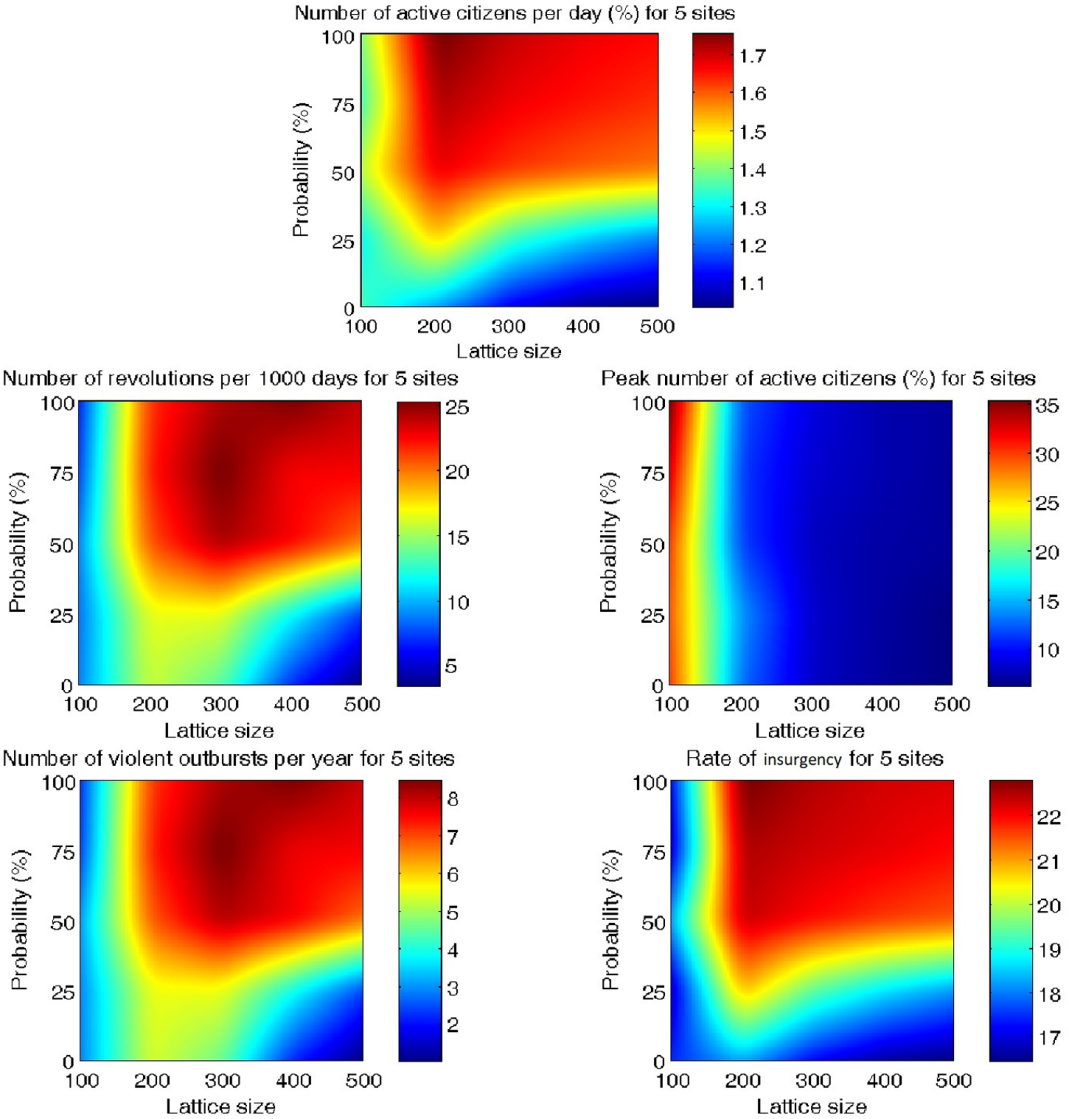
(A) Number of active citizens per day (in percent), (B) number of revolutions per 1,000 days, (C) peak number of active citizens (in percent), (D) number of violent outbursts per year, (E) and rate of insurgency for five preferential gathering sites with respect to the lattice size and probability.

Fig 13 shows some trends with different gathering probabilities and lattice size for the case of five preferential gathering sites. For different numbers of preferential gathering sites and all lattice sizes above 100x100, the number of active citizens per day increases with the increase in the attractiveness probability of the gathering sites. For different numbers of preferential gathering sites and all probabilities above 0.25, the number of active citizens per day at first increases with the increase in the lattice size and then gradually decreases. This is similar to the behavior that is shown above (Fig 11A) in the case of a citizen moving to gathering site with probability 1.

For different numbers of preferential gathering sites the number of revolutions per 1,000 days for smaller lattices is relatively unchanged for non-zero attractiveness probabilities. For bigger lattices, the number of revolutions increases with the increase in the probability, although this increase is the biggest for the case of five preferential gathering sites. With the increase in the number of preferential gathering sites, the clusters of activity become spread around the lattice, which resembles the case with no sites. For different numbers of preferential gathering sites, the number of revolutions per 1,000 days for all probability values at first increases and then decreases for bigger lattices. The increase for smaller lattices and probabilities above 50% and the decrease for bigger lattices for probabilities below 50% are much more substantial in the case of five preferential gathering sites.

The peak number of active citizens is relatively stable with respect to probability. Although the peak number of active citizens significantly decreases for all probabilities with increasing lattice size, it remains above 5% for lattice sizes up to *LD* = 300, which is the threshold for considering an outburst as large-scale. It is worth mentioning that even though the peak number of active citizens stays constant with the increased probability for all lattice sizes, the number of revolutions changes dramatically.

For different numbers of preferential gathering sites, the number of violent outbursts per year behaves in a similar way as the number of revolutions per 1,000 days.

For different numbers of preferential gathering sites, the rate of insurgency for different numbers of preferential gathering sites and all lattice sizes above 100x100 slightly increases with the increase in the attractiveness probability. For all probabilities, the rate of insurgency at first increases for small lattices and then slightly decreases for bigger lattices.

For probabilities 50% and above, the simulation outcomes display similar qualitative behaviors as they do for the case of citizens moving to preferential gathering sites with 100% probability. As the attractiveness probability of the gathering sites decreases, the sites are only weakly attractive and the situation approaches that of having no preferential gathering sites.

### 3.2 Koopman Mode Decomposition

**Fig 14:**
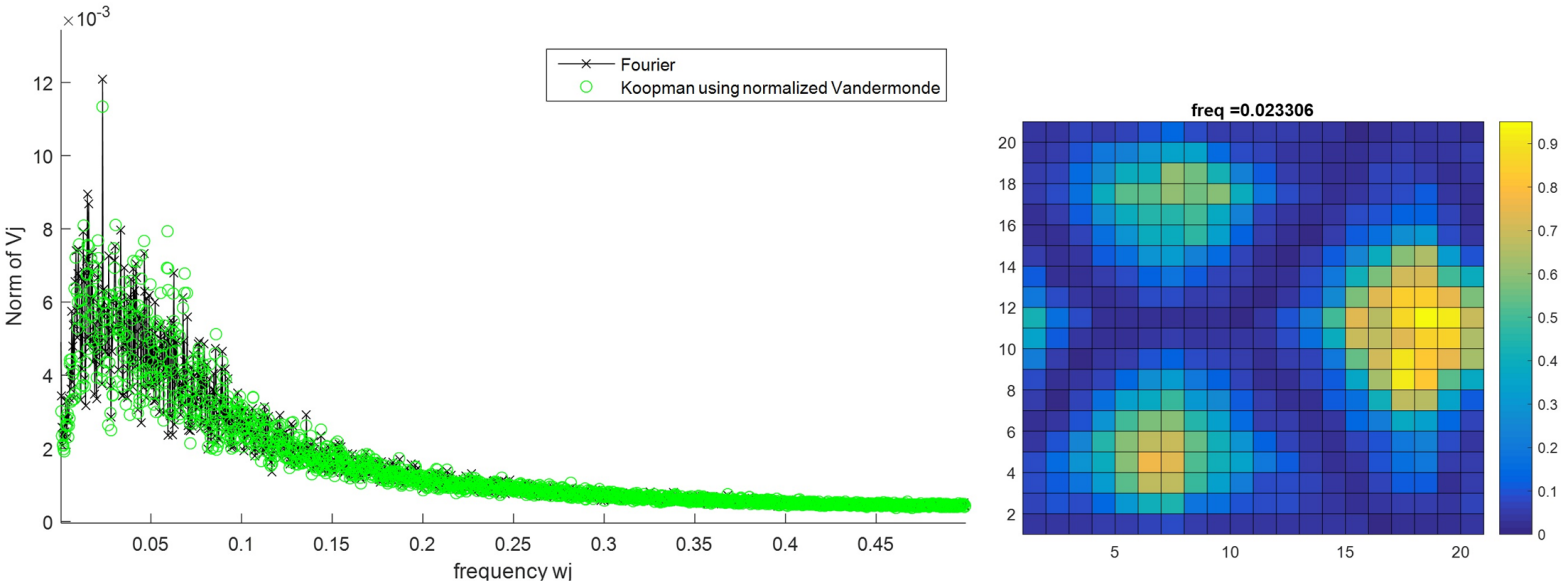
Koopman Mode Decomposition of the number of active citizens for the lattice of size 200x200 for the case with 3 preferential gathering sites (left) and the Koopman Mode with largest norm (right).

**Fig 15:**
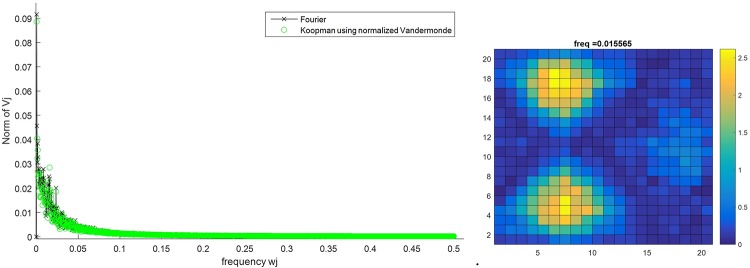
Koopman Mode Decomposition of the number of intimidated citizens for the lattice of size 200x200 for the case with 3 preferential gathering sites (left) and the Koopman Mode with the largest norm (right).

**Fig 16:**
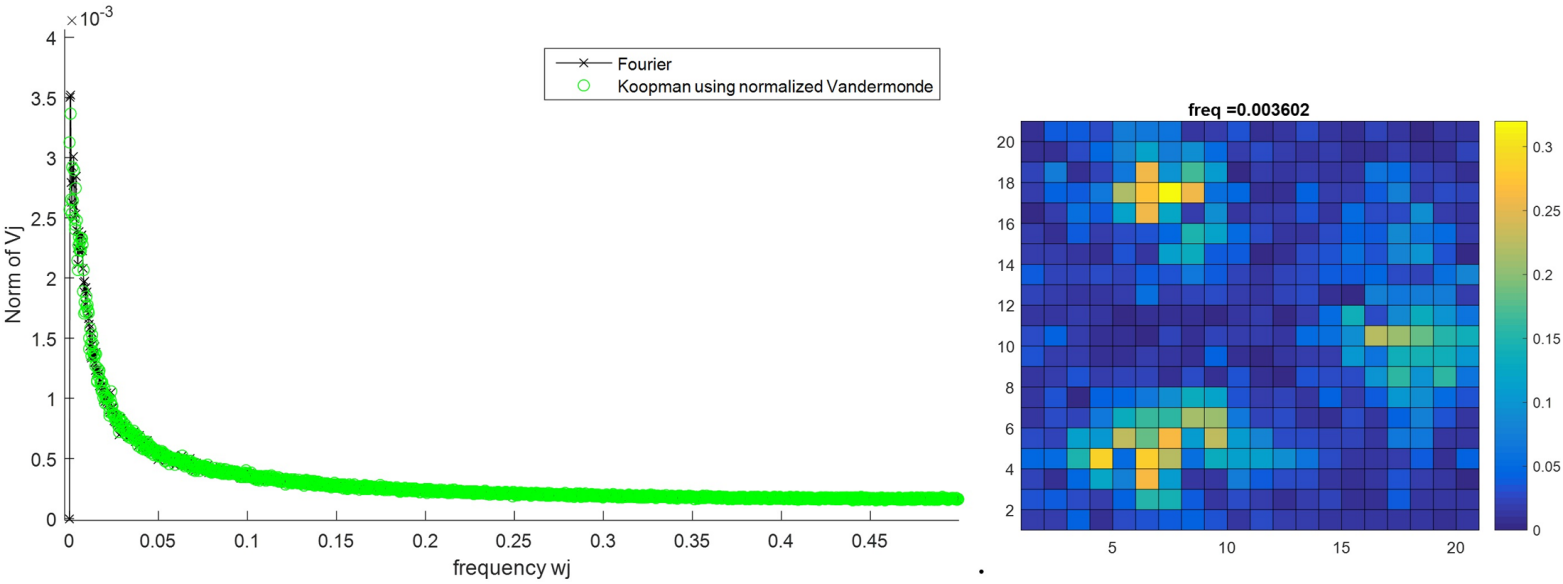
Koopman Mode Decomposition of the number of LEOs for the lattice of size 200x200 for the case with 3 preferential gathering sites (left) and the Koopman Mode with the largest norm (right).

To explore the structure of this complex dynamical model, we calculated the Koopman Mode Decomposition on the spatial distribution of active citizens, intimidated citizens, and LEOs for a 200x200 lattice test case with 3 preferential gathering sites. The grid is divided into 20 cells in each direction and the concentration of each type of population is calculated in each cell. The location of intimidated citizens is their last location as active citizens before intimidation. Figs 14, 15 and 16 show the Fourier spectrum (computed with fft)and the KMD spectrum (see the Arnoldi-type algorithm in [16] for details on computing the KMD spectrum) for the concentration of active citizens, intimidated citizens, and LEOs, respectively, and the real part of the selected Koopman mode. The locations of the three preferential gathering sites are the centers of the agents’ concentrations. The remarkable feature of these plots is that, despite the highly stochastic nature of agent interactions there is a clear spatial and temporal order, evidenced in Koopman Modes, that provides a template for analysis and control.

It is interesting to note that although the LEOs can move quicker than citizens (they make multiple moves on average as compared to citizens), the dominant Koopman mode for the LEOs’ distribution actually has a slower time-dependence than the dominant modes to the active citizens and intimidated citizens (a dominant mode frequency of 0.0036 for LEO’s vs 0.0233 and 0.0156 for active and intimidated citizens, respectively). This is compensated for the fact that the dominant Koopman mode for the LEOs shows that there are less concentrated around the gathering sites as compared to the citizens; the LEOs are distributed throughout the lattice more than citizens, who aggregate strongly to the gathering sites. An interpretation of this is that while citizens move toward the gathering site, there are still a number of citizens that roam between the sites. The LEOs still need to police these areas so that large-scale outbursts do not occur. This in turn requires that a few LEOs move away from the gathering sites in order to cover the areas in between. Thus even though LEOs can move faster than citizens, it is the active and intimidated citizens that have a faster changing, but more spatially localized, distributions. In fact, the distributions for active and intimidated citizens change almost an order a magnitude faster than the distribution for the LEOs does.

## Discussion and conclusions

In previous studies, citizens and LEOs move under random walks, and any local aggregation of citizens leading to a large-scale outburst of insurgency is more due to chance stochastic effects than any regularity or structure in the model. In this paper, we incorporated structure into the ABM model by introducing preferential gathering sites for citizens. This allowed us to study the effect of organized aggregation of citizens on urban insurgency.

For large lattice sizes, large-scale outbursts of activity are relatively rare for both the case of no preferential gathering sites and a large number of gathering sites. The case of no gathering sites can be thought of as the limit of infinitely many gathering sites by the following reasoning. The movement rule for a citizen has them move toward the closest gathering site and pick one randomly if multiple sites are the same distance away. A slight modification of the rule is to say that the citizen chooses to move toward a gathering site in their visual range with probability inversely proportional to the distance they are from the site. In the limit of a dense number of gathering sites, this random walk reduces approximately to the original movement rule for the citizen.

Furthermore, there is a non-monotonic behavior in the number of large-scale outbursts with respect to the number of preferential gathering sites. It is low for one or two gathering sites, peaks at around four or five gathering sites, and then decreases with increasing numbers of lattice sizes. The non-monotonicity is a result of an interesting trade-off in the local concentration of citizens due to the number of gathering sites—the local concentration of citizens drops with increasing numbers of gathering sites—and the decreasing ability of LEOs to adequately cover an increasing number of gathering sites—there are less LEOs per site as the number of sites increase. When there is one or two gathering sites, LEOs can aggregate at the site and are able to intimidate large amounts of citizens quickly, thereby suppressing the active citizens. In other words, if LEOs know that activity only occurs at a few locations (and nowhere else), then the LEOs’ optimal strategy, if their goal is to suppress the number of active citizens, is to just stake out those locations. When there are one or two gathering sites, there is a high local concentration of citizens at those sites (especially for moderate to large lattice sizes) and, in order to keep the number of active citizens low on average, there must be a large number of intimidated citizens. This leads to the situation where a large number of intimidated citizens can re-enter the population in close proximity at close to the same time and then become active due to the large surge in the local density. This surge then leads to a large-scale outburst. While it is true that a moderate number of gathering sites have a larger number of large-scale outbursts than a small number of gathering sites, it is also true that the severity of the outbursts for one or two sites is much greater, as measured by the peak number of active citizens during a large-scale outburst. This is best seen in the moderate to large lattice sizes (*LD* ≥ 300). On the other hand, for a large number of gathering sites and lattice sizes, 300x300 or greater, the number of large-scale outbursts is comparable to having just one gathering site. However, the severity of a large-scale outburst, as measured by the peak number of active citizens during the outburst, is much less.

Thus, this non-monotonicity in the number of large-scale outbursts indicates that a small number of organized units produces a larger insurgency effect than a larger number of distributed units. Furthermore, if the goal is both to suppress the number of large-scale outbursts and the severity of the ones that do occur, it would be a better strategy to offer a large number of gathering sites to citizens rather than restricting the number of places they can assemble. The effect of offering a large number of gathering sites is to distribute the citizens spatially, thereby depressing local concentrations of citizens. The tradeoff for having a larger number of gathering sites and less frequent and less severe large-scale outbreaks is that there will be more active citizens on average.

We have also shown that there exists interesting, quasi-periodic, temporal structure to the dynamics which arises due to the spatial organization induced by the preferential gathering sites. This behavior was revealed by Koopman Mode Analysis of the statistically stationary portions of the time series. A, perhaps initially, surprising outcome was that the distributions of the LEOs varied an order of magnitude slower than the distributions for the active and intimidated citizens even though LEOs can move 4 times faster than citizens. Less surprising was that the distributions for the active and intimidated citizens were strongly localized around the preferential gathering sites. In contrast, the distribution for the LEOs was more spatially distributed and not as localized around the gathering sites. The localization of the citizens around the gathering sites is due to the nature of the ABM model—citizens moved toward a gathering site they see and never move away. Since this is the case, once LEOs move into their optimal positions, there is little need to move away since they can continue to intimidate active citizens near their current locations. The wider spatial distribution of the LEOs is due to the fact that not all citizens see and move to the gathering site; they move in between them in a random walk. A small number of LEOs are needed to patrol those areas in order to suppress large-scale outbursts.

We would like to emphasize that we are not suggesting policy based upon these results. Our goal was to investigate the introduction of environmental morphology leading to organization of insurgency using a simple agent-based model. A second goal was to introduce to the wider community the efficacy of Koopman Mode Analysis in extracting meaningful spatial patterns with coherent temporal behavior from dynamic data.

## Data management

All datasets generated by our model are housed in a Zenodo public repository and can be found at: https://doi.org/10.5281/zenodo.1284618. The datasets possess their own DOI number and can be cited as [26].

## Supporting information

S1 Appendix The agent-based model of urban insurgency. (PDF)

S2 Appendix Model parameters for the case of high rate of insurgency. (PDF)
