# Supplementary material for: An agent-based model of urban insurgence: Effect of gathering sites and Koopman mode analysis
Source: PLoS One. 2018 Oct 5;13(10):e0205259. doi: 10.1371/journal.pone.0205259 (PMC6173437; doi:10.1371/journal.pone.0205259)
Supplement: S1 Appendix — (PDF) [file pone.0205259.s001.pdf]

S1 Appendix: The agent-based model of urban insurgence

Model variables

Each citizen j is assigned the hardship $H(j)$ drawn from the uniform distribution $U(0,1)$. The hardship is heterogeneous across citizens and is constant in time for each citizen. The perceived legitimacy L of the law enforcement authority is equal across citizens, constant in time, and is chosen between 0 and 1. The hardship and the legitimacy are used to define each citizen's grievance $E(i) = H(i) \cdot (1 - L)$. Some citizens are more willing to pursue insurgency than others, encoded in the model by the risk aversion parameter. Each individual's risk aversion $K(i)$ is drawn from $U(0,1)$ and is constant in time for each citizen. The citizen's vision v is a circle of radius v that comprises lattice positions that the citizen is able to inspect. It is equal across citizens. The citizen's perceived net risk $N(i)$ is defined as follows: $N(i) = K(i) \cdot P(i)$, where $P(i)$ is a function of the ratio of LEOs to active citizens in a citizen's vision radius. If for a law-abiding citizen the difference $E(i) - N(i)$ exceeds T, where T is some threshold, then the citizen becomes criminally active. If, for an active citizen, the difference $E(i) - N(i)$ exceeds T, then the citizen stays active. Otherwise, he/she becomes law-abiding. In summary, the citizen's rule for being active or quiescent is the following: If $E - N > T$ be active; otherwise, be quiescent.

The citizen's perceived risk function $P(i)$ is defined as:

$$P(i) = P(C(i)/A(i)) = 1 - exp(-k'(C(i)/A(i))) \sum_{j=0}^{15} \frac{(k'(C(i)/A(i)))^j}{j!},$$

where $A(i)$ is the number of active citizens (including self) within citizen's vision, $C(i)$ is the number of LEOs within citizen's vision, and the constant $k' = 62.6716$ is found from the condition that $P(1/4) = 0.5$. The perceived risk is in fact zero up to a threshold value, after which it increases monotonically, thus giving it a sigmoidal shape. The sigmoidal shape encodes a level of irrational behavior by citizens, where the real risk of being incarcerated is being diminished by the proportion of others in the same situation.

In general, citizens are less likely to engage in insurgency as the local ratio between

number of LEOs and active citizens increases due to a fear of being identified and intimidated. The citizen state (active or quiescent) can be regarded as a function of threshold T (see [1]). In particular, in the case when $T > 1 - L$, all citizens are always quiescent regardless of the lattice situation; we call them “never active” and denote their number as G. In the case when $T < -1$, all citizens are “always active”, we denote their number as R. When $-1 < T < 0$, $G = 0$ and all citizens are either “always active” or “conditionally active” (active or quiescent depending on the lattice situation). The case when $0 < T < 1 - L$, is the most realistic one with all three groups of population present. In practice the threshold T and legitimacy L for the model run can be found using statistical data on fractions of R and G in the population:

$$T = \frac{2R}{1-G}; \tag{1}$$

$$L = 1 - \frac{2R}{G(1-G)}. \tag{2}$$

Movement models and state update rule

Law enforcement operates through the rule that a LEO agent intimidates the nearest active citizen. This rule leads to dynamics in which local crime hotspots attract police action, thus reflecting the modern problem-oriented policing law enforcement strategies. For more information on roots of the model in criminology theory see [1, 2]. The LEOs' vision w is a circle of radius w that comprises lattice positions that the LEO is able to inspect. It is equal across LEOs. The citizens' vision v is a circle of radius v which is also equal across citizens. Citizens and LEOs have a maximum number of moves they can make during a single day; citizens can move once and LEOs can move four times. At the end of the day, citizens and LEOs will have moved and updated their state the maximum number of times. Agents take turns moving on the lattice; however, the order in which they move is random. The state of each citizen can either be active in criminal and/or violent activity, quiescent, or intimidated. We now describe the update procedure for each day.

Randomly select an agent (citizen or LEO) that has not used up their number of daily moves. If the selected person is a citizen, the assigned intimidation term is

checked and, if not zero, both the assigned intimidation term and the number of available moves is decremented by one. No other updates to that citizen are performed. If the intimidation term is zero and the citizen was previously intimidated, he or she is released to the same spot where they were intimidated and their state is set to quiescent; if that spot is occupied, the citizen is released to the nearest unoccupied spot. Citizens move according to the following movement rule. If a citizen is active, then he/she tries to move in his/her Moore neighborhood in the direction of the closest preferential gathering site with a probability $\mathbb{P}$ (in simulations $\mathbb{P}$ is fixed at either 0%, 25%, 50%, 75%, or 100%). If an agent is quiescent, he/she picks a random cell in his/her Moore neighborhood and if the selected cell is unoccupied, he/she moves there, otherwise the agent stays put. The citizens number of available moves is decremented by one. After, the movement phase has been completed, the state of the citizen (quiescent or active) is calculated depending on the difference between its grievance and net perceived risk; if $E(i) - N(i) > T$, then the citizen becomes active, otherwise, the citizen is quiescent.

If the selected person is a LEO, they inspect all sites within their vision radius w. If there is at least one active citizen, the LEO jumps to the location of the nearest active citizen and intimidates the citizen. The citizen becomes intimidated for a specific amount of time and does not influence the lattice situation (it does not occupy its lattice position). The amount of the intimidation time is assigned randomly from $U(0, J_{max})$, where J_{max} is the maximum amount of the intimidation time. If there is no active citizen within w of the LEO, then the LEO picks a random neighboring location on the lattice (from its Moore neighborhood) and, if that location is unoccupied, moves there. If the location is occupied, the LEO stays at its current location. Continue to select agents until every agent has moved their maximum number of times. Once every agent has moved, end the day and reset the number of each agent can make to their maximum.

References

1. Fonoberova M, Fonoberov VA, Mezić I. Global sensitivity/uncertainty analysis for agent-based models. Reliability Engineering & System Safety. 2013;118:8–17.

2. Fonoberova M, Fonoberov VA, Mezic I, Mezic J, Brantingham PJ. Nonlinear dynamics of crime and violence in urban settings. Journal of Artificial Societies and Social Simulation. 2012;15(1):2.
