# Supplementary material for: An agent-based model of urban insurgence: Effect of gathering sites and Koopman mode analysis
Source: PLoS One. 2018 Oct 5;13(10):e0205259. doi: 10.1371/journal.pone.0205259 (PMC6173437; doi:10.1371/journal.pone.0205259)
Supplement: S2 Appendix — (PDF) [file pone.0205259.s002.pdf]

S2 Appendix: Model parameters for the case of high rate of insurgency

We set the citizen density at 0.7 (i.e. citizens occupy 70% of the lattice); number of LEOs per 1,000 citizens is 1.27; citizen vision is 14; LEO vision is 14; LEO speed is 4 (i.e. LEOs move 4 times per day); maximum intimidation term is 120 days; fraction of "always active" citizens R = 0.025; fraction of "never active" citizens G = 0.45; fraction of "conditionally active" citizens is 0.525. Threshold T and legitimacy L can be calculated by using formulas (1) and (2) from S1 Appendix. All "always active" citizens are initially intimidated.

Table 1. Model Parameters for the case of high rate of insurgency.

**Parameter Name**	**Parameter Value**
citizens density	0.7 (i.e. citizens occupy 70% of the lattice)
number of LEOs per 1,000 citizens	1.27
citizen vision	14
LEO vision	14
LEO speed	4 (i.e. LEOs move 4 times per day)
maximum intimidation term	120 days
fraction of "always active" citizens R	0.025
fraction of "never active" citizens G	0.45
fraction of "conditionally active" citizens	0.525
